# Quantifying adherence to growth hormone treatment: the easypod™ connect observational study (ECOS)

**DOI:** 10.1186/1687-9856-2013-S1-P46

**Published:** 2013-10-03

**Authors:** Peter Davies, Ho-Seong Kim, Martin Borkenstein, Minlian Du, Jeremy Kirk, Ludmila Kostalova, Jan Lebl, Sandro Loche, Andrea Luczay, Marc Nicolino, Svante Norgren, Dolores Rodriguez Arnao, John Vandermeulen, Christoph Gasteyger, Jürgen Zieschang, Monia Zignani

**Affiliations:** 1 University of Queensland, Brisbane, Australia; 2 Yonsei University, Seoul, Korea; 3 Med.Univ.Graz, Graz, Austria; 4 First affiliated Hospital of Sun Yat-sen University Guangzhou, China; 5 Birmingham Children's Hospital, Birmingham, UK; 6 Comenius University Medical School, Bratislava, Slovakia; 7 Charles University in Prague, Prague, Czech Republic; 8 Ospedale Microcitemico - ASL Cagliari, Cagliari, Italy; 9 Semmelweis University, Budapest, Hungary; 10 Lyon University, Lyon, France; 11 Karolinska University Hospital, Stockholm, Sweden; 12 Hospital Universitario Gregorio Marañón, Madrid, Spain; 13 McMaster Children's Hospital and McMaster University, Ontario, Canada; 14 Merck Serono S.A, Geneva, Switzerland; 15 Merck KGaA, Darmstadt, Germany

## 

Recombinant human growth hormone (r-hGH) is indicated for pediatric patients with a variety of growth disorders. Until recently, analysis of adherence to treatment has been limited by recall bias and reliance on self-reporting. Accurate recorded data on r-hGH use can now be collected using the easypod™ auto-injector. The multinational easypod™ connect observational study (ECOS) was launched in 2010 to collect and analyze r-hGH dosing, clinical and auxological data from patients prescribed r-hGH via easypod™. Twelve countries are currently recruiting patients.

The primary objective is to assess adherence in patients receiving r-hGH via easypod™. Secondary objectives include describing the impact of adherence on clinical outcomes and identifying adherence patterns.

Data will be obtained from patients’ medical notes and uploaded from auto-injectors. Auxological parameters are collected, and prescribed dosing data recorded at clinic visits as per routine clinical practice. Annual adherence will be calculated (number of days the patient administered injections divided by the expected number of injection days over 1 year, as a percentage). Dose intensity (total amount of dose received divided by planned amount of dose over 1 year, as a percentage) will be analyzed. Adherence data will be correlated with clinical outcomes. An adherence pattern will also be developed based on patients’ age, sex, indication, self-injection, and time on treatment. The study will run until 2015, with yearly analyses, and will be overseen by a multinational scientific steering committee.

With data from ECOS, it will be possible to accurately assess r-hGH treatment adherence in various growth disorders and explore its potential impact on growth. Ultimately, drivers of and barriers to treatment adherence will be identified, allowing appropriate support programs to be developed.

